# Uptake Patterns of [^18^F]Fluoroestradiol PET/MRI in Benign Breast Lesions and Molecular Breast Cancer Subtypes

**DOI:** 10.3390/cancers18040696

**Published:** 2026-02-20

**Authors:** Thomas Spiegel, Sazan Rasul, Nina Pötsch, Alexander Stiglbauer-Tscholakoff, Panagiotis Kapetas, Lukas Nics, Paulina Gebhart, Ivo Rausch, Zsuzsanna Bago-Horvath, Paola Clauser, Pascal A. T. Baltzer, Marcus Hacker, Thomas H. Helbich, Katja Pinker

**Affiliations:** 1 Department of Biomedical Imaging and Image-Guided Therapy, Division of General and Pediatric Radiology, Medical University of Vienna, Waehringer Guertel 18-20, 1090 Vienna, Austria; thomas.spiegel@meduniwien.ac.at (T.S.);; 2 Department of Biomedical Imaging and Image-Guided Therapy, Division of Nuclear Medicine, Medical University of Vienna, Waehringer Guertel 18-20, 1090 Vienna, Austria; sazan.rasul@meduniwien.ac.at (S.R.);; 3 Department of Obstetrics and Gynecology, Comprehensive Cancer Center, Medical University of Vienna, Waehringer Guertel 18-20, 1090 Vienna, Austria; 4 Center for Medical Physics and Biomedical Engineering, Medical University of Vienna, Waehringer Guertel 18-20, 1090 Vienna, Austria; 5 Department of Pathology, Medical University of Vienna, Waehringer Guertel 18-20, 1090 Vienna, Austria; 6 Department of Radiology, Breast Imaging Service, Memorial Sloan Kettering Cancer Center, 300 E 66th Street, New York, NY 10065, USA

**Keywords:** breast cancer, molecular imaging, ^18^F-Fluoroestradiol, benign breast lesions, molecular subtypes, PET/MRI

## Abstract

**Background/Objectives:** Estrogen receptor (ER) expression is a key biomarker in breast cancer (BC) and guides endocrine therapy selection. Estrogen receptor-targeted imaging with 16ɑ-[^18^F]-fluoro-17β-estradiol (^18^F-FES) PET is recommended in several clinical guidelines for noninvasive assessment of ER status. In clinical practice, ^18^F-FES PET may also identify ER-negative malignancies or benign breast lesions with variable uptake patterns. This study aimed to systematically characterize ^18^F-FES PET/MRI uptake patterns in benign breast lesions and across breast cancer subtypes defined by receptor status, histology, and molecular phenotype. **Methods:** This retrospective single-center study included 41 women with 50 breast lesions who underwent simultaneous ^18^F-FES PET/MRI prior to any treatment. Histopathology or long-term follow-up served as the standard of reference. Maximum and mean standardized uptake values (SUVmax and SUVmean) were derived using MRI-based lesion delineation. **Results:** Both benign and malignant breast lesions measuring < 10 mm demonstrated low ^18^F-FES uptake (SUVmax < 1.00). ^18^F-FES uptake among benign breast lesions was variable, with SUVmax ranging from 0.44 to 1.57. In contrast, ER-positive lesions ≥ 10 mm exhibited substantially higher ^18^F-FES uptake (median SUVmax 2.76; range 1.23–9.74) compared with ER-negative tumors of similar size (SUVmax 0.30–0.94). ^18^F-FES uptake was consistent across histologic BC subtypes and did not differ significantly among ER-positive molecular subtypes. No significant associations were observed with HER2 status or tumor grade. **Conclusions:** Awareness of the heterogeneous ^18^F-FES uptake patterns in benign breast lesions, as well as the limited sensitivity for detecting ER-positive tumors < 10 mm, is essential for accurate image interpretation. ^18^F-FES PET/MRI enables reliable assessment of ER expression in BC lesions ≥ 10 mm, with uptake patterns remaining consistent across molecular and histologic subtypes.

## Simple Summary

Hormone receptor status, in particular estrogen receptor (ER) status, plays a pivotal role in therapeutic decision-making in breast cancer. Positron emission tomography (PET) using a radiolabeled estrogen analog [^18^F]Fluoroestradiol (^18^F-FES) enables noninvasive, whole-body assessment of ER expression in vivo. In this retrospective analysis, we systematically evaluated imaging characteristics of breast lesions on ER-targeted PET/MRI to elucidate patterns of tracer uptake in benign lesions and across molecular breast cancer subtypes. All small breast lesions < 10 mm exhibited low ^18^F-FES uptake. Notably, a subset of benign lesions demonstrated tracer uptake overlapping with ER-positive malignancies, highlighting a potential diagnostic pitfall. ER-positive tumors ≥ 10 mm were characterized by high tracer uptake, whereas ER-negative tumors of comparable size consistently showed low uptake values. Overall, ^18^F-FES uptake patterns were concordant across molecular and histologic breast cancer subtypes. These findings provide clinically relevant insights that may enhance the interpretation of ER-targeted breast imaging and support its integration into clinical practice.

## 1. Introduction

Breast cancer (BC) is the most frequently diagnosed malignancy and remains a leading cause of cancer-related mortality among women worldwide [1]. The majority of BCs express estrogen receptors (ER), with rates ranging between 79% and 85%, although these proportions vary according to factors such as age, menopausal status, tumor grade, and histologic subtype [2,3,4,5]. ER-positive tumors are likely to respond to endocrine therapy, which substantially improves progression-free and overall survival [6]. Accordingly, ER assessment is essential for selecting systemic therapy and guiding individualized management.

ER status for therapy guidance is routinely determined from tissue samples from core needle biopsy using immunohistochemistry. Tissue sampling from biopsy is always subject to selection bias, reflects only a small portion of a lesion and cannot fully capture inter- and intratumoral heterogeneity of ER expression [7,8,9,10]. In the metastatic setting, biopsy of a metastatic lesion may not be feasible, and treatment decisions have to rely on the ER status of the primary tumor, which may not represent current receptor expression, potentially leading to suboptimal or even inappropriate therapy [8,10,11].

In addition, immunohistochemistry detects all ER but does not distinguish between functionally active and inactive receptors, and not all tumors classified as ER-positive respond to ER-targeted treatment [10,12]. These limitations highlight the need for noninvasive imaging approaches capable of comprehensively assessing functional ER expression of the entire primary tumor and the metastatic disease.

16ɑ-[^18^F]-fluoro-17β-estradiol (^18^F-FES) is a radiolabeled estrogen analog that enables rapid, noninvasive whole-body assessment of functionally active ER [10]. ^18^F-FES positron emission tomography (PET) correlates strongly with ER immunohistochemistry and reliably detects ER-positive lesions [13]. Several guidelines support its use as an adjunct to biopsy, particularly for (i) guiding endocrine therapy decisions at the initial diagnosis of metastatic disease or following progression on prior therapy; (ii) assessing ER status in challenging or inaccessible biopsy sites or when biopsy is non-diagnostic; and (iii) clarifying inconclusive findings from conventional imaging. ^18^F-FES PET may also be appropriate for staging invasive lobular carcinoma (ILC) and low-grade invasive ductal carcinoma (IDC), evaluating malignancies of unknown primary, assessing extra-axillary nodal and distant metastases, and detecting recurrent or metastatic lesions [10,12,14,15,16]. While prior studies have mainly focused on ^18^F-FES PET for assessing ER-positive lesions and guiding therapy [10,16,17,18,19,20], data on unexpected or discordant findings remain limited.

In the staging setting, ^18^F-FES PET/CT or PET/MRI detects not only different types of breast cancer but also benign lesions, which demonstrate different uptake patterns. Here, we performed a retrospective analysis, which systematically assessed a spectrum of breast lesions, including those not typically expected to show ^18^F-FES uptake. Our aim was to provide insights into ^18^F-FES uptake patterns across receptor status, histologic and molecular BC subtypes and benign breast lesions.

## 2. Materials and Methods

### 2.1. Patients

This retrospective analysis was conducted using ^18^F-FES PET/MRI data prospectively acquired as part of a single-center study on prediction and assessment of neoadjuvant treatment response in BC. Patients underwent simultaneous multiparametric ^18^F-FES PET/MRI of the breast and whole body before and during therapy. Written informed consent was obtained from all participants, and the study was approved by the Institutional Ethics Committee of the Medical University of Vienna (EK 510/2009).

**Figure 1:**
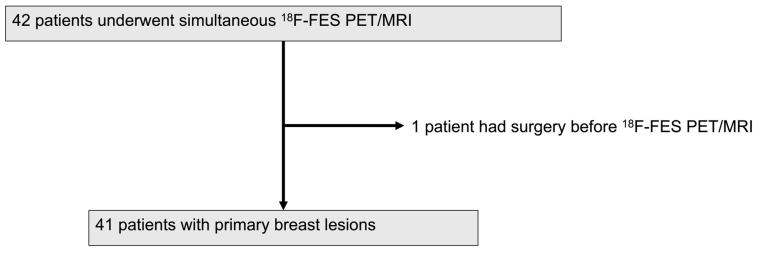
Flow chart of patient inclusion.

For the retrospective analysis, 42 female patients who underwent ^18^F-FES PET/MRI for the evaluation of suspicious breast lesions (BI-RADS 4: suspicious; BI-RADS 5: highly suggestive of malignancy) prior to any treatment were identified [21]. The final cohort included 41 patients with 50 breast lesions imaged between March 2021 and July 2022 (Figure 1).

### 2.2. ^18^F-FES PET/MRI Acquisition Protocol

Simultaneous multiparametric ^18^F-FES PET/MRI was performed using a Biograph mMR system (Siemens Healthineers, Erlangen, Germany), which integrates a 3.0 Tesla MRI scanner with an MRI-compatible PET detector [22].

Patients received an injection of 2 MBq per kilogram of bodyweight ^18^F-FES, and PET/MR imaging commenced approximately 60 ± 10 min after tracer administration. Total scanning time was approximately 60 min. MRI-based attenuation correction was performed using the standard Dixon-based attenuation correction approach as implemented in software version V11P.

PET data were collected with a 3D acquisition mode providing an axial field of view of ~26 cm and a transverse field of view of 59 cm, yielding a system sensitivity of 13.2 cps/kBq. Static PET images were reconstructed using ordinary Poisson 3D ordered-subset expectation maximization (OP-OSEM) with 3 iterations and 21 subsets, employing all routine corrections (normalization, randoms, scatter, attenuation, and decay), and applying a Gaussian filter (4 mm FWHM) to reduce noise.

Multiparametric MRI was acquired using a dedicated 16-channel breast coil (Rapid Biomedical, Würzburg, Germany). The protocol consisted of (i) axial T2-weighted imaging: TR/TE = 4820/192 ms; matrix 640 × 480; FOV 360 × 360 mm; slice thickness 2.5 mm; interslice gap 3 mm; flip angle 128°; (ii) diffusion tensor imaging (DTI) using a 2D diffusion-weighted single-shot spin-echo echo-planar sequence with parallel imaging and fat suppression: TR/TE = 4500/87 ms; matrix 190 × 112; FOV 212 × 360 mm; slice thickness 4 mm; gap 5.2 mm; flip angle 90°; diffusion encoding in 12 directions with b = 0 and 800 s/mm^2^; and (iii) dynamic contrast-enhanced (DCE) MRI: gadolinium-based contrast agent (Dotarem, 0.1 mL/kg body weight, Guerbet, Villepinte, France) was used. For T1 mapping, five pre-contrast axial T1 VIBE sequences with flip angles of 2°, 10°, 20°, 30°, and 40° were acquired. Subsequently, T1 TWIST dynamic imaging was performed: TR/TE = 4.7/1.3 ms; matrix 352 × 352; FOV 440 × 440 mm; slice thickness 2 mm; no gap; flip angle 10.5°; 23 phases at 14 s temporal resolution. Subtraction images were generated for interpretation.

For this retrospective analysis, only the prone breast acquisitions were used.

### 2.3. Radiotracer

16ɑ-[^18^F]-fluoro-17β-estradiol was commercially supplied by Curium Austria GmbH (Hausmannstätten, Austria). Production and quality control were performed according to Good Manufacturing Practice (GMP), and all batches met predefined release criteria for clinical use [23].

### 2.4. Image Analysis

^18^F-FES PET/MR images of the breasts were analyzed using open-source LIFEx software (version 6.30; https://www.lifexsoft.org (accessed on 17 February 2026)) [24]. Breast lesions were identified on dynamic contrast-enhanced (DCE) postcontrast subtracted images. Lesion size was measured as the maximum diameter in the axial plane. Tumor delineation was performed on the DCE-MRI sequence of the simultaneously acquired PET/MRI examination, as DCE provides superior contrast and reliable visualization of tumor margins compared to ^18^F-FES PET, especially in lesions with low uptake.

Acquisition of PET and DCE-MRI data was performed simultaneously on a hybrid PET/MRI scanner, ensuring intrinsic spatial co-registration. The alignment between PET and MRI datasets was visually verified in axial, coronal, and sagittal planes to confirm accurate co-registration prior to image analysis. The postcontrast phase with the highest lesion conspicuity was selected. A three-dimensional volume of interest (VOI) was defined for each lesion by semiautomatic segmentation, exploiting the high contrast between lesions and surrounding breast tissue [25]. The DCE-MRI-based VOI was applied to the PET dataset to obtain maximum and mean standardized uptake values (SUVmax and SUVmean).

**Figure 2:**
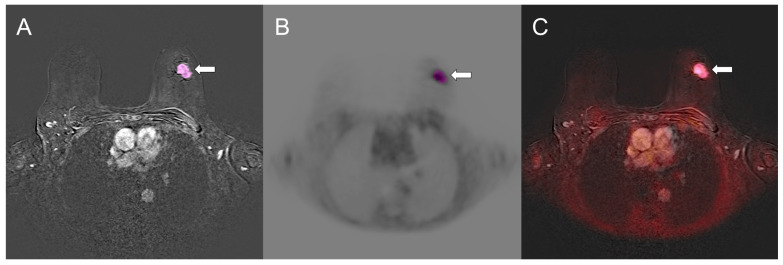
Volume of interest (VOI) placement in a 79-year-old woman with an ER-positive, G2, luminal B-like invasive ductal carcinoma (ER 80%, PR 30%, HER2 negative, Ki-67 40%) in the left breast (white arrows). (**A**) Semiautomated segmentation was performed on postcontrast subtracted dynamic contrast-enhanced MRI to generate a 3D-VOI encompassing the enhancing mass with indistinct borders (pink). (**B**) Co-registered ^18^F-FES PET images and (**C**) fused PET/MRI images show marked radiotracer uptake corresponding to the enhancing lesion. The VOI defined on DCE-MRI was applied to the co-registered PET dataset for extraction of quantitative PET metrics (SUVmax 4.22; SUVmean 1.83).

Because ^18^F-FES uptake was low in many lesions, PET-based threshold segmentation methods (fixed cutoffs, %SUVmax thresholds, or background-corrected methods adapted from ^18^F-FDG PET [26]) were unsuitable for consistent quantification. MRI-based delineation was therefore used to ensure full morphological coverage and reproducible SUV measurements. The methodological workflow is illustrated in Figure 2.

Background activity was measured as the SUVmean of spherical VOIs (1 cm^3^) placed in the thoracic aorta and in normal breast parenchyma of the same breast, avoiding lesions, skin, and large vessels.

All images were analyzed by a resident radiologist under the supervision of a breast radiologist and a nuclear medicine physician with more than 30 and 12 years of experience, respectively, all trained in hybrid imaging. SUVs were normalized to the injected dose and body weight.

### 2.5. Histopathological Assessment

Histopathology served as the standard of reference. For benign lesions that did not undergo biopsy, a follow-up of more than 2 years served as the standard of reference [27]. Core biopsy samples obtained at inclusion and surgical specimens were analyzed to determine tumor histology, grade, and immunohistochemical status—including estrogen receptor (ER), progesterone receptor (PR), Ki-67 expression, and overexpression and/or amplification of human epidermal growth factor receptor 2 (HER2). ER positivity was defined as nuclear staining ≥ 1% of tumor cells [4,28]. HER2 was considered positive if staining intensity was 3+ (strong) or 2+ (moderate) with positive subsequent in situ hybridization (ISH) [28]. The St. Gallen surrogate molecular subtype definitions were used to classify breast lesions [29], with a Ki-67 cutoff of ≥20% used to define high proliferation. Either biopsy results or postoperative histopathology served as the standard of reference for lymph node (LN) metastasis.

### 2.6. Statistical Analysis

Descriptive statistics were used to summarize the data. Continuous variables are presented as medians with interquartile range (IQR) and range. Categorical variables are expressed as counts and percentages. Normality of continuous variables was assessed using the Shapiro–Wilk test and visual inspection of histograms. Because most variables were non-normally distributed and contained outliers, between-group comparisons were performed using the Mann–Whitney U test for two groups and the Kruskal–Wallis test for comparisons involving more than two groups. Receiver operating characteristic (ROC) curve analysis was conducted to evaluate diagnostic performance, expressed as the area under the curve (AUC). Correlations were evaluated using Spearman’s rank coefficients. All statistical tests were two-sided, and exact *p*-values < 0.05 were considered statistically significant; all *p*-values were interpreted exploratorily. Statistical analyses were performed using IBM SPSS Statistics, version 30.0.0.0 (IBM Corp., Armonk, NY, USA).

## 3. Results

### 3.1. Patient Characteristics

A total of 41 patients (median age 53 years, IQR 41–67, range 28–86) with a total of 50 breast lesions (median size 18.8 mm, IQR 13.1–28.5 mm, range 7.0–70.0) were included. Patient and lesion characteristics are summarized in Table 1.

^18^F-FES PET/MRI uptake characteristics of benign and malignant breast lesions are summarized in Table 2.

**Table 2:** Lesion size, SUVmax, SUVmean, and background activity of normal breast parenchyma and thoracic aorta among subgroups of breast lesions ≥ 10 mm. Note: Data are presented as medians and ranges. Statistical comparison results are presented in Table 3. Interquartile ranges (IQR) are provided in Supplementary Table S1.

	n	Lesion Size (mm)	Lesion SUVmax	Lesion SUVmean	Breast Parenchyma SUVmean	Thoracic Aorta SUVmean
Benign	7	13.1 (10.1–26.3)	0.93 (0.72–1.57)	0.72 (0.50–1.02)	0.41 (0.14–0.50)	1.93 (1.11–2.47)
ER-positive BC	34	24.7 (11.0–70.0)	2.76 (1.23–9.74)	1.51 (0.74–4.89)	0.24 (0.07–1.06)	1.48 (0.76–5.56)
Luminal A-like	5	14.4 (12.1–19.5)	2.13 (1.33–3.58)	1.23 (0.74–2.21)	0.27 (0.08–0.36)	1.55 (1.27–2.43)
Luminal B-like	29	27.0 (11.0–70.0)	2.89 (1.23–9.74)	1.51 (0.77–4.89)	0.21 (0.07–1.06)	1.47 (0.76–5.56)
Luminal B-like HER2+	3	31.0 ^†^ (27.5–32.0)	3.35 ^†^ (2.19–3.98)	1.86 ^†^ (1.30–2.20)	0.49 ^†^ (0.20–0.98)	1.54 ^†^ (1.47–1.93)
ER-negative BC (TNBC)	3	18.4 ^†^ (14.4–35.7)	0.89 ^†^ (0.30–0.94)	0.57 ^†^ (0.17–0.60)	0.20 ^†^ (0.17–0.28)	1.41 ^†^ (1.36–1.89)
ER-positive IDC	30	23.5 (11.0–42.3)	2.76 (1.23–9.74)	1.51 (0.74–4.89)	0.25 (0.07–1.06)	1.49 (0.76–5.56)
ER-positive ILC	4	58.8 ^†^ (36.1–70.0)	4.03 ^†^ (2.36–5.73)	1.96 ^†^ (1.25–2.41)	0.15 ^†^ (0.14–0.54)	1.46 ^†^ (1.44–1.84)
ER-positive G1	5	14.4 (12.1–34.7)	2.13 (1.33–3.63)	1.23 (0.74–1.51)	0.27 (0.12–0.36)	1.55 (1.30–2.43)
ER-positive G2	19	27.0 (11.0–70.0)	2.98 (1.23–7.25)	1.61 (0.77–3.70)	0.20 (0.07–1.06)	1.47 (0.76–5.56)
ER-positive G3	10	24.8 (15.2–60.2)	2.76 (1.70–9.74)	1.56 (1.00–4.89)	0.22 (0.12–0.98)	1.49 (0.96–1.93)
ER-positive/LN metastasis	20 *	29.5 (11.0–70.0)	3.47 (1.70–9.74)	1.75 (0.93–4.89)	0.20 (0.08–0.98)	1.48 (0.96–5.56)
ER-positive/LN-benign	13 *	22.6 (12.1–29.9)	2.13 (1.33–4.22)	1.24 (0.74–1.85)	0.33 (0.07–1.06)	1.55 (0.76–2.47)

^†^ Median reported for small sample size. * One patient with LN metastasis had bifocal BC; only the index lesion was included (n = 33). Abbreviations: ER, estrogen receptor; BC, breast cancer; TNBC, triple-negative breast cancer; IDC, invasive ductal carcinoma; ILC, invasive lobular carcinoma; LN, lymph node.

**Table 3:** *p*-values and AUC for statistical comparisons of imaging parameters.

Groups Compared	Parameter	Test	*p*-Value	AUC
ER-positive BC vs. benign lesions	SUVmax	Mann–Whitney U	**<0.001**	0.983
	SUVmean	Mann–Whitney U	**<0.001**	0.962
Luminal A-like vs. Luminal B-like BC	SUVmax	Mann–Whitney U	0.196	0.690
	SUVmean	Mann–Whitney U	0.177	0.697
ER-positive vs. ER-negative (TNBC) ^‡^	SUVmax	Mann–Whitney U	**<0.001**	-
	SUVmean	Mann–Whitney U	**<0.001**	-
Tumor Grade (G1, G2, G3)	SUVmax	Kruskal–Wallis	0.428	-
	SUVmean	Kruskal–Wallis	0.132	-
ER-positive/LN metastasis vs. ER-positive/LN-benign	SUVmax	Mann–Whitney U	**0.006**	-
	SUVmean	Mann–Whitney U	**0.008**	-
	Lesion size	Mann–Whitney U	**0.018**	-

Notes. Statistically significant *p*-values (*p* < 0.05) are shown in bold. **^‡^** Small sample size; TNBC, n = 3. Abbreviations: ER, estrogen receptor; BC, breast cancer; LN, lymph node.

Of the 50 lesions, 42 were BCs and eight were benign. One patient had a bifocal BC and one a bilateral BC of identical histology. Five patients with BC had an additional benign breast lesion, probable fibroadenomas based on their image characteristics, and demonstrated long-term stability of at least two years, indicative of benign etiology. Three suspicious breast lesions, classified as BI-RADS 4, underwent core needle biopsy, yielding fibroadenomas or fibroadenomatous hyperplasia, which were benign and concordant with imaging.

### 3.2. Benign Breast Lesions

**Figure 3:**
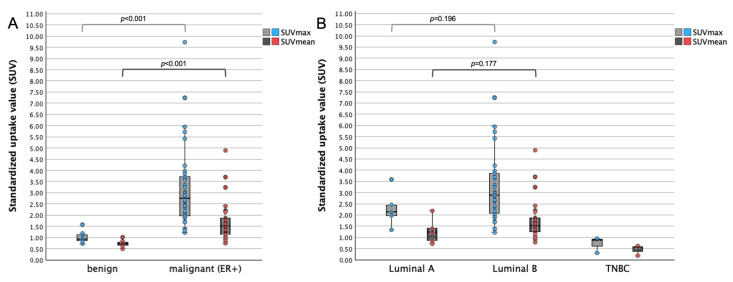
Scattered box plots of maximum and mean standardized uptake values (SUVmax and SUVmean) measured on ^18^F-FES PET/MRI in breast cancers and benign lesions ≥ 10 mm. Boxes represent the interquartile range (IQR), horizontal lines indicate medians, whiskers extend to 1.5 × IQR, and individual points denote outliers. *p*-values shown in the figure were calculated using the Mann–Whitney U test for subgroups with n ≥ 5. (**A**) SUVmax and SUVmean in benign lesions versus ER-positive breast cancers. ER-positive cancers show higher uptake values, although partial overlap is observed. (**B**) SUVmax and SUVmean across molecular subtypes. ER-positive (Luminal A and Luminal B) and ER-negative (TNBC; n = 3) BC differed significantly in SUVmax and SUVmean (*p* < 0.001 and *p* < 0.001, respectively; Table 3).

**Figure 4:**
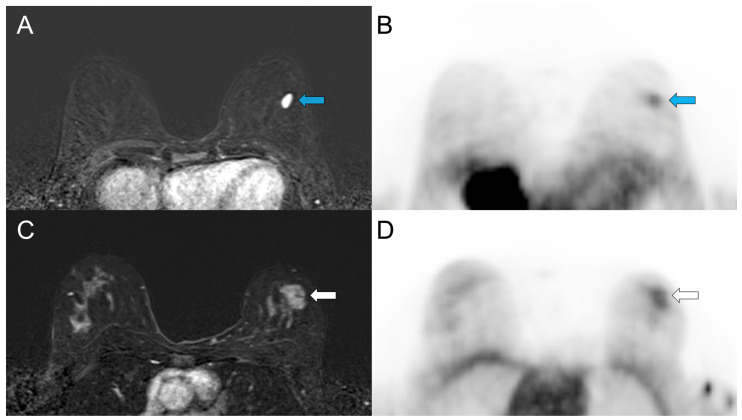
^18^F-FES PET/MRI of the breast in two patients with benign breast lesions. Subtracted dynamic postcontrast T1-weighted MR images (**A**,**C**) and corresponding ^18^F-FES PET images (**B**,**D**). (**A**,**B**) A 46-year-old woman with known fibroadenoma in the left breast stable in shape and size for more than two years (blue arrows). MRI shows oval, circumscribed, homogeneous enhancing mass. ^18^F-FES SUVmax is 1.18 and SUVmean is 0.73. (**C**,**D**) A 50-year-old woman with biopsy-proven benign fibroadenoma in left breast (white arrows). MRI shows irregular, partly indistinct mass with non-enhancing septa. ^18^F-FES SUVmax is 1.57 and SUVmean is 1.02.

Benign breast lesions (n = 8) had a median size of 13.0 mm (IQR 10.2–14.1, range 7.2–26.3). SUVmax ranged from 0.44 to 1.57 (median 0.92, IQR 0.75–1.16), and SUVmean ranged from 0.31 to 1.02 (median 0.70, IQR 0.53–0.81). Benign breast lesions ≥ 10 mm (n = 7) demonstrated significantly lower SUVmax and SUVmean than ER-positive BCs ≥ 10 mm (*p* < 0.001 and *p* < 0.001, AUC = 0.983 and AUC = 0.962, respectively; Figure 3A). Three of seven (42.9%) benign breast lesions ≥ 10 mm showed ^18^F-FES uptake with SUVmax ranging from 1.08 to 1.57, comparable to those reported in ER-positive BCs (Figure 3 and Figure 4).

### 3.3. Malignant Breast Lesions

Among the 42 BCs, 39 (92.9%) were ER-positive and 3 (7.1%) were ER-negative. All five ER-positive BCs < 10 mm (three Luminal A, two Luminal B) showed low ^18^F-FES uptake with SUVmax < 1.00.

ER-positive BCs ≥ 10 mm (n = 34, 81%) showed SUVmax ranging from 1.23 to 9.74 (median 2.76, IQR 1.98–3.75) and SUVmean ranging from 0.74 to 4.89 (median 1.51, IQR 1.12–1.93). All three ER-negative BCs ≥ 10 mm showed low uptake, with SUVmax ranging from 0.30 to 0.94, and SUVmean ranging from 0.17 to 0.60. ER-positive IDCs had similar median SUVmax and SUVmean in comparison to ER-positive ILCs (Table 2 and Table 3).

**Figure 5:**
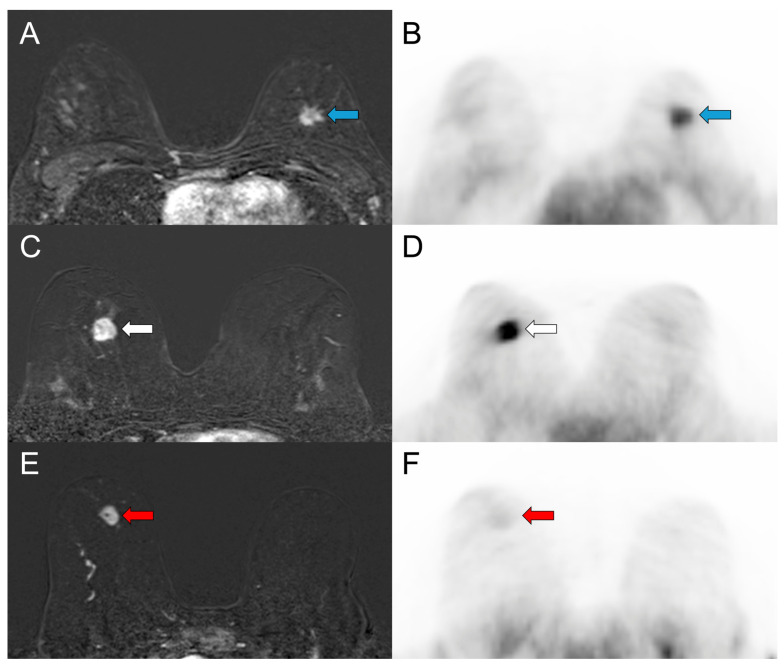
^18^F-FES PET/MRI of the breast in three patients with invasive ductal carcinoma, illustrating ER-status and radiotracer uptake. Subtracted dynamic postcontrast T1-weighted breast MR images (**A**,**C**,**E**) and corresponding ^18^F-FES PET images (**B**,**D**,**F**) are shown. (**A**,**B**) Images of a 46-year-old woman with an ER-positive, G1, Luminal A-like invasive ductal carcinoma (ER 90%, PR 90%, HER2 negative, Ki-67 10%) in the left breast (blue arrows). MRI shows an irregular, spiculated, heterogeneous mass. ^18^F-FES PET SUVmax is 2.13 and SUVmean is 1.23. (**C**,**D**) Images of a 35-year-old woman with an ER-positive, G3, Luminal B-like invasive ductal carcinoma (ER 90%, PR 10%, HER2 negative, Ki-67 60%) in the right breast (white arrows). MRI shows an irregular, partly indistinct, heterogeneous mass. ^18^F-FES SUVmax is 3.04 and SUVmean is 1.85. (**E**,**F**) Images of a 66-year-old woman with a triple-negative, G3, invasive ductal carcinoma (Ki-67 60%) in the right breast (red arrows). MRI shows an oval, partly heterogeneous mass with low ^18^F-FES uptake (SUVmax 0.89, SUVmean 0.57) in PET, consistent with the absence of ER expression.

Luminal B-like BCs (n = 29) tended to have higher uptake values with median SUVmax of 2.89 and median SUVmean of 1.51 in comparison to Luminal A-like BCs (n = 5) with median SUVmax of 2.13 and median SUVmean of 1.23 (*p* = 0.196 and *p* = 0.177, respectively; Figure 3B). Figure 5 shows representative ^18^F-FES PET/MR images of ER-positive (Luminal A, Luminal B) and ER-negative BCs.

SUVmax and SUVmean of Luminal B-like HER2-positive BCs (n = 3) were within the range of Luminal B-like HER2-negatives. ER-positive BCs ≥ 10 mm, SUVmax and SUVmean showed a moderate correlation with tumor size (r = 0.40, *p* = 0.021 and r = 0.46, *p* = 0.007). ER expression was weakly correlated with SUVmax and SUVmean (r = 0.27, *p* = 0.105 and r = 0.29 and *p* = 0.085). SUVmax and SUVmean showed no significant difference among tumor grades (*p* = 0.428 and *p* = 0.132, respectively). In ER-positive BCs ≥ 10 mm, 20 cases (60.6%) had LN metastasis, while 13 cases (39.4%) did not. ER-positive BCs ≥ 10 mm with LN metastasis demonstrated significantly higher SUVmax and SUVmean than those without LN involvement (*p* = 0.006 and *p* = 0.008, respectively; Table 2 and Table 3).

## 4. Discussion

In this study, we provide insights into ^18^F-FES uptake patterns in benign breast lesions and across receptor status, histologic and molecular BC subtypes using an integrated PET/MRI scanner. Notably, ^18^F-FES PET uptake was observed in both benign and malignant breast lesions, with uptake values being influenced by lesion size. ^18^F-FES uptake was higher in ER-positive than in ER-negative BCs. Consistent ^18^F-FES uptake was observed across molecular and histologic BC subtypes.

In fact, size is crucial in PET quantification, especially for smaller lesions, where physical limitations of the scanner reduce measured activity. The underestimation of small lesions in PET is primarily explained by partial volume effects. In our cohort, benign and malignant breast lesions smaller than 10 mm demonstrated low ^18^F-FES uptake (SUVmax < 1.00) and lesion size correlated moderately with uptake values. This aligns with findings of previous studies, including Chae et al. [30] and Gemignani et al. [31], with reduced sensitivity for smaller lesions. Thus, there is a need for advanced techniques such as partial volume correction and deep learning algorithms, which mathematically correct for partial volume effects to improve the accuracy of small lesion detection. On the other hand, all lesions < 10 mm that may be missed on an ^18^F-FES PET scan could be assessed on DCE-MRI. This emphasizes that the integration of morphologic and kinetic MRI information remains essential to detect and characterize breast lesions, even when low-level tracer uptake is observed. This rationale, where PET images provide molecular information while MRI examination contributes high-resolution morphological and functional information [32], aligns with recommendations from several societies for the use of PET/MRI in BC staging [15].

It has to be noted that information regarding ^18^F-FES uptake characteristics of benign breast lesions is scarce. Dehdashti et al. [33] reported no ^18^F-FES uptake in benign breast lesions ≥ 10 mm. In contrast, in our cohort, benign breast lesions ≥ 10 mm had SUVmax values ranging from 0.72 to 1.57. Notably, in three out of seven lesions (42.9%), SUVmax ranged from 1.08 to 1.57, values comparable to some ER-positive BCs. ER-positive cells and ER expression have been reported in benign breast lesions, including fibroadenomas, as in our cohort [34,35]. Since ^18^F-FES is a radiolabeled estradiol analog that binds to ER in all lesions expressing ER, both benign and malignant tumors may demonstrate tracer uptake. This likely explains the observed ^18^F-FES uptake in some benign breast lesions in this study, probably fibroadenomas based on MRI morphology and enhancement kinetics. This is an observation that is important for the interpretation of tracer activity of breast lesions seen on staging ^18^F-FES PET/MRI in clinical practice.

Furthermore, ^18^F-FES uptake in BCs was concordant with ER status and consistent with the literature [30,36]. All ER-positive BCs ≥ 10 mm showed consistently higher uptake values (median SUVmax 2.76, range 1.23–9.74) in comparison to ER-negative BCs ≥ 10 mm, which demonstrated an SUVmax < 1.00. These findings are in line with prior studies reporting minimal tracer accumulation in ER-negative disease and confirming that ^18^F-FES uptake is strongly linked to ER availability [13,30]. This supports the role of ^18^F-FES PET/MRI as a reliable noninvasive method for the assessment and quantification of ER expression in BC, particularly in lesions with a size ≥ 10 mm.

In our cohort, ^18^F-FES uptake values were similar among common ER-positive histologic and molecular BC subtypes. Standardized uptake values of Luminal A-like and Luminal B-like BCs did not differ significantly, reflecting that ^18^F-FES PET primarily captures ER expression, heterogeneity, and availability, rather than differences in proliferation defined by Ki-67 that distinguish Luminal A from Luminal B. Consistent with prior observations by Gemignani et al. [31], HER2-status and tumor grade did not appear to influence ^18^F-FES uptake. ER-positive ILCs showed consistently high uptake values within the range of IDCs. These findings are in line with prior studies reporting high ^18^F-FES uptake in ER-positive ILCs. Overall, our findings support the reliability of ^18^F-FES PET across ER-positive molecular and histologic BC subtypes, including ILCs, regardless of proliferation rate, HER2-status, or tumor grade.

ER expression did not correlate significantly with ^18^F-FES standardized uptake values in our cohort, with uptake values varying widely even among BCs with similar ER expression. This is consistent with Takahashi et al. [37]. These findings are explained by several factors: ^18^F-FES PET reflects whole-lesion ER status and functionally active ligand-binding receptors, whereas immunohistochemistry from biopsy samples represents only a limited tumor region [12,30]. Additionally, variations in stromal content and partial volume effects may further influence measured uptake values [37]. Thus, ^18^F-FES PET provides functional information about ER status beyond static immunohistochemical measurements.

Our study has some limitations. First, the single-center setting may reduce external validity. Second, sample sizes for several subgroups were small, which may have reduced statistical power, restricted subgroup analyses, and limited generalizability. Although systematic assessment of ^18^F-FES uptake across receptor status and histologic and molecular BC subtypes remains limited to our relatively small patient cohort, this study is the first to describe observed uptake patterns in benign breast lesions, which are frequent incidental findings in staging PET/MRI.

The lack of Allred-scoring may have limited the assessment of correlations between ER expression and ^18^F-FES uptake. However, given the uniformly high ER expression in this cohort and the score’s dependence on the percentage of ER-positive cells, any potential variability in scoring would likely have been minimal [38].

MR-based attenuation correction may introduce variability in standardized uptake values, potentially limiting direct comparisons with PET/CT-based studies. Nevertheless, PET/MRI has been validated for quantitative analyses, with strong correlations between PET/CT and PET/MRI uptake parameters reported [32].

## 5. Conclusions

^18^F-FES PET/MRI provides robust ER assessment in BC lesions ≥ 10 mm and demonstrates consistent uptake across common histologic and molecular subtypes of ER-positive disease. Uptake values in benign and malignant breast lesions < 10 mm were low. The observation of ^18^F-FES uptake in benign breast lesions (3/7, 42.9%), which are frequent incidental findings in staging PET/MRI, underscores that tracer accumulation is not entirely specific to ER-positive BCs. Consequently, accurate interpretation of PET findings requires careful correlation with MRI, complementary imaging modalities, and histopathological results to reliably distinguish benign from malignant breast lesions, even in cases of low-level tracer uptake and in lesions smaller than 10 mm.

As ER-targeted PET imaging becomes increasingly integrated into routine clinical practice, a detailed understanding of physiological and pathological ^18^F-FES uptake patterns is critical to minimize diagnostic misclassifications and optimize clinical decision-making.

## Figures and Tables

**Table 1:** Patient and lesion characteristics.

Patients	Number	%
Premenopausal	20	48.8
Postmenopausal	21	51.2
Total	41	100
**Histological type**	**Number of lesions**	**%**
**Benign lesions**		
Fibroadenoma	7	14.0
Fibroadenomatous atypical hyperplasia (FAH)	1	2.0
**Breast cancers**		
Invasive ductal carcinoma (IDC)	36	72.0
Invasive lobular carcinoma (ILC)	5	10.0
Ductal carcinoma in situ (DCIS)	1	2.0
Total	50	100
**Molecular subtype (BC)**	**Number of lesions**	**%**
Luminal A-like	8	19.0
Luminal B-like/HER2−	28	66.7
Luminal B-like/HER2+	3	7.1
Triple-negative (TNBC)	3	7.1
Total	42	100
**Tumor grade (BC)**	**Number of lesions**	**%**
Grade 1	8	19.0
Grade 2	20	47.6
Grade 3	13	31.0
High-grade DCIS	1	2.4
Total	42	100

Abbreviations: FAH, fibroadenomatous atypical hyperplasia; IDC, invasive ductal carcinoma; ILC, invasive lobular carcinoma; DCIS, ductal carcinoma in situ; BC, breast cancer; HER2, human epidermal growth factor receptor 2; TNBC, triple-negative breast cancer.

## Data Availability

Research data are available from the Corresponding Authors by reasonable request.

## Supplementary Materials

The following supporting information can be downloaded at: https://www.mdpi.com/article/10.3390/cancers18040696/s1, Table S1: Interquartile ranges (IQR) of lesion size, SUVmax, SUVmean, and background activity of normal breast parenchyma and thoracic aorta in breast lesions ≥ 10 mm.
