# Supplementary material for: Uptake Patterns of [18F]Fluoroestradiol PET/MRI in Benign Breast Lesions and Molecular Breast Cancer Subtypes
Source: Cancers (Basel). 2026 Feb 20;18(4):696. doi: 10.3390/cancers18040696 (PMC12939227; doi:10.3390/cancers18040696)
Supplement: Supplementary file 1 [file cancers-18-00696-s001.zip › cancers-4150256-supplementary.docx]

Supplementary Material

**Table S1.** Interquartile ranges (IQR) of lesion size, SUVmax, SUVmean, and background activity of normal breast parenchyma and thoracic aorta in breast lesions ≥10 mm.

|  | **n** | **Lesion size (mm)** | **Lesion**  **SUVmax** | **Lesion**  **SUVmean** | **Breast parenchyma**  **SUVmean** | **Thoracic aorta**  **SUVmean** |
| --- | --- | --- | --- | --- | --- | --- |
| Benign | 7 | 10.4-14.3 | 0.82-1.18 | 0.62-0.83 | 0.20-0.50 | 1.28-1.93 |
| ER-positive BC | 34 | 15.4-32.0 | 1.98-3.75 | 1.12-1.93 | 0.14-0.36 | 1.27-1.90 |
| Luminal A-like | 5 | 12.9-18.0 | 1.65-3.02 | 0.81-1.81 | 0.16-0.36 | 1.39-2.16 |
| Luminal B-like | 29 | 17.3-33.4 | 2.03-3.92 | 1.19-2.00 | 0.14-0.37 | 1.25-1.88 |
| ER-positive IDC | 30 | 15.1-28.5 | 1.95-3.65 | 1.03-1.85 | 0.16-0.36 | 1.26-1.92 |
| ER-positive G1 | 5 | 12.9-27.1 | 1.65-3.04 | 0.81-1.46 | 0.18-0.36 | 1.41-2.16 |
| ER-positive G2 | 19 | 16.5-32.0 | 1.89-3.86 | 1.04-2.14 | 0.14-0.37 | 1.27-2.18 |
| ER-positive G3 | 10 | 15.4-32.6 | 2.16-4.34 | 1.26-2.25 | 0.17-0.39 | 1.09-1.83 |
| ER-positive / LN-metastasis | 20 * | 17.2-36.9 | 2.43-5.07 | 1.31-2.21 | 0.14-0.33 | 1.29-1.89 |
| ER-positive / LN-benign | 13 * | 14.5-25.45 | 1.65-2.91 | 0.96-1.56 | 0.21-0.37 | 1.18-2.12 |

**Notes.** IQR is only reported for subgroups with n ≥ 5. Median and range are provided in Table 2.

***** One patient with LN-metastasis had bifocal BC; only the index lesion was included (n = 33).

**Abbreviations:** ER, Estrogen receptor; BC, Breast cancer; IDC, Invasive ductal carcinoma; LN, Lymph node.
